# Exploring the Interplay Between Adversity, Neurocognition, Social Cognition, and Functional Outcome in People With Psychosis: A Narrative Review

**DOI:** 10.3389/fpsyt.2021.596949

**Published:** 2021-03-08

**Authors:** Victoria Rodriguez, Monica Aas, Natasha Vorontsova, Giulia Trotta, Romayne Gadelrab, Navneet Kaur Rooprai, Luis Alameda

**Affiliations:** ^1^Department of Psychosis Studies, Institute of Psychiatry, Psychology and Neuroscience, King's College London, London, United Kingdom; ^2^NORMENT Centre for Psychosis Research, Oslo University Hospital, University of Oslo, Oslo, Norway; ^3^Division of Mental Health and Addiction, Department of Mental Health Research and Development, Vestre Viken Hospital Trust, Drammen, Norway; ^4^Department of Psychology, Institute of Psychiatry, Psychology and Neuroscience, King's College London, London, United Kingdom; ^5^Social, Genetic and Developmental Psychiatry Centre, Institute of Psychiatry, King's College London, London, United Kingdom; ^6^Service of General Psychiatry, Treatment and Early Intervention in Psychosis Program, Lausanne University Hospital (CHUV), Lausanne, Switzerland; ^7^Departamento de Psiquiatría, Centro Investigación Biomedica en Red de Salud Mental (CIBERSAM), Instituto de Biomedicina de Sevilla (IBIS), Hospital Universitario Virgen del Rocío, Universidad de Sevilla, Sevilla, Spain

**Keywords:** childhood adversity, functional outcome, neurocognition, social cognition, narrative review

## Abstract

History of adversity is associated with subsequent psychosis, and with a spectrum of cognitive alterations in individuals with psychosis. These cognitive features go from neurocognitive aspects as working memory and attention, to complex social cognitive processes as theory of mind and emotional perception. Difficulties in these domains impact patients' social and occupational functioning, which has been shown to be more impaired in those previously exposed to childhood trauma. However, the interplay between adversity, neurocognition, and functioning is yet poorly understood. This narrative review aims to explore the evidence on whether deficits in neurocognitive and social cognitive domains may act as possible putative mechanism linking adversity with functioning in people with psychosis. We show available evidence supporting the link between adversity and poorer functioning in psychosis, especially in chronic stages; and replicated evidence suggesting associations of social cognition and, to a lesser extent, neurocognition with impairment in functioning in patients; although there is still an important gap in the literature testing particularly deficits in social cognition as mediator of the link between adversity and functional decline in psychosis. Targeting interventions focusing on neurocognition and social cognition in individuals with adversity and psychosis seems important, given the severe deterioration of these patients in these domains, although more research is needed to test whether such treatments can specifically improve functioning in individuals with psychosis and adversity. Literature aiming to understand the determinants of functional outcome should consider the pervasive impact of childhood adversity, and its related effects on cognition.

## Introduction

Psychotic disorders are among the leading causes of disability worldwide (1) with recovery rates in terms of functional level below 15% of the patients with schizophrenia (2). Functional outcome, which covers activities of daily living, vocational activities, social relationships, and degree of independence (3), is a key element of the poor outcome in psychotic disorders, greatly impacting the social disability burden (4). Deficit in functional level is detectable before the onset of the illness, present in its early stages, and it often persists, remaining relatively poor despite resolution of acute psychosis (5). Moreover, evidence suggests some independence of the functional decline from symptom dimensions such as delusions, hallucinations, and disorganization (6). Thus, finding potentially treatable determinants of functional outcome is one of the main goals in schizophrenia research (7, 8).

Both Neurocognition and Social Cognition are also very important domains in psychosis (9–12), and such deficits account for the diversity of functional outcomes in the disorder, more effectively than symptoms (7, 13, 14). Interventions such as cognitive remediation (8), Social Cognition and Interaction Training (SCIT) (15), Social Cognitive Skill Training (SCST) (16) or metacognitive and social cognition training (MSCT) (17), among others (18, 19), have been developed in order to improve such domains, with promising benefits (20). However, despite these observed benefits, whether they have a positive impact on functional outcomes is still unclear (18). Meta-analytic evidence has shown that three-quarters of variance in functional outcome remains to be explained (7), which suggests that other factors also have an impact on functioning.

Childhood adversity affects functioning and cognition in the general population (21, 22), and these domains have been shown also to be more impaired in patients with previous exposure to childhood adversity (21, 23). Evidence suggests that some cognitive biases and neurocognitive domains mediate the link between adversity and psychosis (24, 25). In this regard, Howes and Murray developed a sociodevelopmental-cognitive model, providing an integrated explanation of how the social environment can lead to psychosis through neurobiological changes in the brain as well as cognitive bias (26). Moreover, a recent systematic review has shown that negative schemas about the self, the world and others mediate considerably the adversity-psychosis association (25). However, these works have not covered which potential mechanisms may operate on the link between adversity and functional decline in those with psychotic disorder. A better understanding of the nature of the association between adversity and functional decline, as well as its interplay with neurocognition and social cognition, may help to better define patients at risk of developing such deleterious outcomes and to specifically apply interventions that can target possible mediating mechanisms. Moreover, whether traumatized individuals with psychosis may better benefit from interventions commonly addressed to improve neurocognition or social cognition (8, 18, 27, 28) remains an intriguing unexplored question.

In this review we will explore the interplay between adversity, neurocognition, social cognition, and functional outcome in people with psychosis. To explore this question we will summarize the relevant evidence on the association between adversity and neurocognition (section Adversity and Neurocognition) overviewing the literature on possible biological pathways in this relationship (section Possible Biological Pathways Involved in Cognitive Deficits); explore available evidence on the relationship between adversity and social cognition (section Adversity and Social Cognition); and how neurocognition and social cognition interplay for their impact on functioning in subjects with psychosis (section Interplay Between Neurocognition, Social Cognition, and Functioning in People With Psychosis). We will appraise evidence exploring the link between adversity and functioning in patients (section Adversity and Functional Outcome), and we will explore emerging evidence suggesting possible mediating pathways between adversity and functioning outcome through cognitive domains (section Is There Evidence of a Mediation Between Adversity and Functional Outcome Through Cognition?). Lastly, we will discuss potential clinical implications of current research, as well as methodological issues and gaps in the literature (section Discussion, Future Directions, and Implications).

## Adversity and Neurocognition

According to the NIMH-Measurement and Treatment Research to Improve Cognition in Schizophrenia (MATRICS), eight different domains of cognitive impairment have been identified for schizophrenia (9): speed of processing, attention and vigilance, verbal learning, working memory, problem solving, visual learning, and social cognition. In the current work, we will refer to the first seven when talking about neurocognition, and discuss social cognition independently.

Childhood adversity, which occurs during a neurodevelopmental period critical for brain maturation and development, has been linked to earlier pruning, reduced gray matter volume, flexibility impairments, and lower IQ in adulthood (21, 29). We know from both animal and human studies that exposure to extreme stress and trauma during periods of brain development are characterized by lasting changes in brain functioning (30, 31) and in the biological stress system such as the Hypothalamic Pituitary Adrenal (HPA) axis (32, 33). Although biological evidence in the field of trauma and psychosis has focused mainly on positive symptoms or psychosis itself, research has attempted to study possible biological mechanisms linking adversity and cognitive functions.

An increasing body of evidence supports that exposure to early life adversity may affect neurocognition (NC) at presentation in psychosis. A recent meta-analysis consisting of 3,315 individuals with a psychotic illness found a significant negative association (with low effects) between overall cognition and childhood adversity, *r* = −0.055, 95% CI −0.09, −0.02. Furthermore, when dividing into subdomains of neurocognition, a modest, negative association was observed between childhood trauma and working memory, *r* = −0.091, 95% CI −0.15, −0.03 (34). As suggested by Vargas et al., an association seems to be present although with low effects, and a careful mapping of different types of childhood adversities, timing of the trauma and severity of exposure is important to drive this field forward (34). One of the few studies that has investigated the association between different types of childhood adversities and cognitive domains found that physical neglect, followed by physical abuse were the strongest predictors of cognitive impairment in psychosis (35). This is supported by a recent independent study by Mørkved et al., which also demonstrated that childhood physical neglect more than other types of trauma were associated with cognitive impairment in adulthood (36). Another important factor is the timing of trauma, which has been found to play an important role on how adversity can increase the risk of psychosis (37), although more studies are needed. For instance, MRI data highlight the importance of specific time of trauma exposure on brain development, given the different processes the brain undergoes between childhood, adolescence, and young adulthood, including periods of production and pruning of synapses and signaling mechanisms (38). Furthermore, it has been reported that reduction in hippocampal volume is associated with childhood sexual abuse at ages 3–5 years and ages 11–13 years, whilst exposure to a stressful event between 14 and 16 years activates the prefrontal cortex (PFC) and is associated with synaptic loss by young adulthood (39). Nonetheless, whether neuroanatomical findings linked to trauma have neurocognitive correlates should still be addressed in future studies.

## Possible Biological Pathways Involved in Cognitive Deficits

Although the biological mechanisms linking adversity with the neurocognitive alterations in patients with psychosis are yet to be fully understood, some biomarkers have been proposed. These studies mainly assess the moderating effect of some biological measures on the association between adversity and cognition in people with psychosis.

The role of cortisol and a dysregulation of the Hypothalamic Pituitary Adrenal (HPA) axis (32, 33) has been extensively studied, with abundant preclinical evidence suggesting that stress increases glucocorticoid secretion, which reduces neurogenesis and synaptogenesis, especially in the hippocampus (40). A putative idea of mechanism could be that trauma is associated with higher levels of glucocorticoids in the brain, leading to a reduction in the number of glucocorticoid receptors in the hippocampus which may reduce the negative feedback from the hippocampus to the HPA axis (41). This results in stress sensitivity, which involves an HPA axis that is over-active and excessively reactive to subsequent environmental stressors, and which further augments glucocorticoid levels (41, 42). There is evidence suggesting that this elevation of glucocorticoids generate neurotoxicity and atrophy in the hippocampus, which may possibly explains the diminution of the size of the hippocampus of patients exposed to adversity (31, 43); as well as their relationship with neurocognitive dysfunction (34).

Brain-derived neurotrophic factor (BDNF) is another important protein for brain development and its low presence may be responsible for the observed reduced plasticity in patients with severe mental disorders (44, 45). A history of childhood adversity or being a met carrier of the BDNF val66met are both associated with a significant reduction of BDNF mRNA levels (46, 47). For example, a study by Aas et al., found that met carriers of the BDNF val66met who reported high levels of childhood trauma (specifically sexual or physical abuse) had reduced volumes of hippocampal subfield CA2/3 and CA4 dentate gyrus compared to patients without childhood trauma and compared to Valine (val/val) carriers (46). Patients who were met carriers and who reported childhood trauma also had the poorest cognitive functioning (48), supporting a role of BDNF levels, childhood trauma, and brain functioning in psychosis. The study is also an example of a two hit model including both environmental and genetic factors targeting the same biological pathway associated with cognitive impairment in psychosis.

Another biomarker suggested to modify the role of trauma on brain development is oxidative stress (49). The study by Alameda et al., found that patients with a higher oxidation status measure in blood was negatively associated with hippocampal volume in those early psychosis patients with trauma, while those with trauma and a lower oxidation status displayed better cognitive functions (specially memory, vigilance/attention, and speed of processing). Thus, as suggested by the authors, a redox profile, characterized by high vs. low oxidation status may represent an important biomarker for defining treatment strategies in traumatized patients with psychosis (49).

Despite this emerging evidence, no clear biological mediating pathway has been consistently explored. Different non-competing biological pathways may be involved and be differentially expressed across individuals with the disorder. Selecting patients based on specific biomarker profiles may allow better capturing the link between adversity and specific neurocognitive domains. Yet, this complex link is far from being fully understood, which makes it difficult to address specific pharmacological means in patients with cognitive impairment (see section Discussion, Future Directions, and Implications).

## Adversity and Social Cognition

Within the social cognition (SC) domain, NIMH consensus recognized five subdomains including: Theory of Mind (ToM), social perception, social knowledge, emotion perception and processing, and attributional style (50). Briefly, ToM involves the ability to infer one's own and other people's mental states (51). Social knowledge refers to awareness of the roles, rules, and goals that characterize social situations and guide social interactions (52), and social perception indicates the ability to judge these roles, rules, and relationships in a social context (53). Emotional recognition is measured as the accuracy at recognizing the emotions of others. Attributional style refers to an individual's tendency to see events as being caused by the self, other individuals or external factors (54). Metacognition is another, broader, social cognitive concept overlapping with the above categories, and is defined as the awareness and understanding of one's own and others' mental processes (55).

Despite the available literature exploring the links between childhood adversity and neurocognitive domains in people with psychosis (34), less attention has been paid to the impact of adversity on social cognition. Associations between different forms of adversity and domains of social cognition have been reported in the general population (56, 57) and in non-psychotic disorders (58), but to the best of our knowledge only 13 studies have addressed this question in samples of individuals with psychosis. Table 1 shows available studies examining the association between abuse or neglect and social cognition domains in people with psychosis. These papers have all been published from 2016 onwards, except one in 2011 (62) which shows the growing amount of interest in this field in recent years. As a whole, seven studies contained analyses with at least one significant association between a category of abuse and a social cognitive domain (24, 60–62, 66, 70, 71); six with a category of neglect (24, 64, 65, 68–70); two with a composite category of adversity (59, 64), and only one study didn't find any association between adversity and social cognition (67). Regarding time of exposure, only one study examined this, showing that neglect at age 11–12 was the strongest predictor of deficits of emotion regulation and mentalizing abilities (69).

**Table 1:** Characteristics of the studies examining the association between abuse or neglect and social cognition domains in people with psychosis.

**Study**	**Country**	**Participants *N* (% female)**	**Age Mean**	**Measures of childhood adversity**	**Cognitive measures**	**Functional outcome measures**	**Main findings**
Aas et al. (59)	Norway	101 SMI (45%)	31.9	CTQ	Brain activation measured with fMRI during presentation of faces with negative or positive emotional expressions	GAF	Stronger differentiation in brain responses between negative and positive faces with higher levels of trauma
Aydin et al. (60)	Turkey	35 SCZ (37.1%) 35 HC (60%)	29.91 SCZ 31.05 HC	CTQ	MAS-A	/	Childhood emotional abuse was related to metacognitive capacity
Brañas et al. (61)	Spain	62 SCZ (46.8%)	31.15	Semi-structured interview	HT; DFAR	/	Patients with childhood trauma other than sexual abuse were more able to recognize fear as a facial emotion
Choi et al. (62)	USA	143 SMI (51%)	38.47	Adapted subscale of the childhood maltreatment assessment scale of (63)	HT; I-SEE	NOSIE-30	The adverse effects of the severity of history of child physical abuse on social functioning were compensated for by greater social inference and lower external locus of control
Garcia et al. (64)	Spain	79 EP (39.2%) 58 HC (48.3%)	25.34 EP 23.95 HC	CTQ	MCCB	GAF	Childhood trauma was associated with poorer social cognition
Kilian et al. (65)	South Africa	56 FEP (25%) 52 HC (33%)	23.8 FEP 25.1 HC	CTQ	MCCB	/	The association between neglect and social cognition was present and was not illness-specific
Lysaker et al. (66)	US	101 SZ (15.2%)	46.26	TAA	MAS; BLERT; WCST; WAIS-III; HVLT; CPT-II	/	Patients with a history of childhood sexual abuse had lower awareness of other people's emotions
Mansueto et al. (24)	Netherlands	757 SMI (25%)	27.66	CTQ	WLT; CPT-HQ; WAIS-III; HT	/	In male psychotic patients, lower mentalization, attention and vigilance mediated the association between childhood neglect and negative symptoms, disorganization, and excitement, while poor working memory mediated association between childhood abuse and disorganization, excitement, and emotional distress
Palmier-Claus et al. (67)	UK	20 SZ (35%) 20 FEP (20%) 14 UHR (57.1%) 120 HC (70.8%)	39.6 SZ 24.6 FEP 22.6 UHR 20.1 HC	CTQ	HT; RMET	PSP	Childhood adversity significantly predicted worse social functioning, but greater in the non-clinical compared to the clinical sample
Rokita et al. (68)	Ireland	74 SZ (32.4%) 116 HC (44.8%)	44.6 SZ 35.0 HC	CTQ	HT; RMET; ERT; WAIS-III	/	Association between physical neglect and emotion recognition in both groups
Schalinski et al. (69)	Germany	168 SMI (33.3%) 50 HC (44%)	27.9 SMI 26.8 HC	MACE	MCCB	/	Cumulative adverse childhood experiences and physical neglect at age 11 were significantly negatively associated with social cognition in patients
Trauelsen et al. (70)	Denmark	92 non-affective FEP (27.2%)	22.4	CTQ	MAS	/	Different types of childhood trauma were associated with better metacognitive abilities
Weijers et al. (71)	Netherlands	87 non-AP (35.6%)	31.7	CECA	HT	SFS	The severity of parental abuse was associated with mentalizing impairment, but not with social dysfunction

*SMI, severe mental illness; CTQ, Childhood Trauma Questionnaire; GAF, Global Assessment of Functioning Scale; fMRI, functional magnetic resonance imaging; SCZ, schizophrenia; HC, healthy control; MAS, Metacognition Assessment Scale; HT, Hinting Task; DFAR, Degraded Facial Affect Recognition; I-SEE, Inventory for Self-Efficacy and Externality; NOSIE-30, Nurses' Observation Scale for Inpatient Evaluation Total Positive Subscale; EP, early psychosis; MCBB, MATRICS Consensus Cognitive Battery; SZ, schizophrenia or schizoaffective disorder; TAA, Trauma Assessment for Adults; BLERT, Bell-Lysaker Emotion Recognition; WCST, Wisconsin card sorting test; WAIS-III, Wechsler adult intelligence scale III; HVLT, Hopkins verbal learning test; CPT-II, Conners Continuous Performance Test II; WLT, World Learning Task; CPT-HQ, Continuous Performance Test; PSP, Personal and Social Performance Scale; RMET, Reading the Mind in the Eyes Task; ERT, Emotion Recognition Task; FEP, First Episode Psychosis; UHR, Ultra High Risk; MACE, Maltreatment and Abuse Chronology of Exposure Scale; non-AP, non-affective psychosis; SFS, Social Functioning Scale*.

Nonetheless, the high levels of heterogeneity in the measures used preclude pointing at specific effects between childhood adversity types and social cognitive subdomains. Furthermore, samples were fairly small, with just one sample above 200 cases (24); and only one study was conducted in FEP (70). Moreover, concerns have been raised with regards to the validity of psychometric properties of existing measures in social cognitions domains in people with psychosis, suggesting an urgent need to improve such instruments (72, 73).

We can conclude that there is some emerging evidence suggesting a link between exposure to abuse, neglect and a dysfunction of various social cognitive domains, but research is still limited and needs consistent replication in large samples.

## Interplay Between Neurocognition, Social Cognition, and Functioning in People With Psychosis

Consistent evidence has accumulated during the last 20 years suggesting the presence of an association between neurocognition and social cognition with functional outcome in people with psychosis (7, 14, 74). Individuals who are able to comprehend social and emotional stimuli may have acquired better interpersonal and communication skills, and thus have a better functional capacity. On the other hand, greater problems in the storage and processing of information in memory and in the ability to think flexibly about abstract ideas results in greater difficulties in thinking about and recognizing emotions (66).

The last meta-analysis in this field, conducted on 166 studies and 12,868 participants has revealed that the association between neurocognition, social cognition and functional outcome shows small-to-medium effect sizes, with 7.3% of the overall variance in functional outcome explained by social cognition, against 4.4% for neurocognition (7). In line with others (75, 76), this work suggests a possible partial mediation between neurocognition and functioning through deficits in social cognition (7), indicating that neurocognition deficits may precede the latter. Despite these relevant findings, it remains to be understood which are the other determinants of the deficits of functioning in patients with psychosis, since a great amount of variance remains unexplained (6). For example, it remains to be explored whether cognitive bias such as negative schemas about the self the world and others, or jumping to conclusions may play a role in the adversity-functional decline dyad in those with psychosis. Moreover, the effect sizes of the associations between neurocognition, social cognition, and functioning being small to medium could mean that deficits in social cognition or neurocognition may be particularly deleterious for specific groups of patients. This suggests the importance of better understanding which subgroups of patients have greater risk to develop social cognition and neurocognition deficits and subsequently poorer functional outcomes, being those patients exposed to trauma potentially among those more vulnerable subgroups.

## Adversity and Functional Outcome

In the last 20 years, childhood adversity has been studied as another potential factor predisposing to functional decline in people with psychosis. Lysaker et al. showed in 2001 for the first time that a history of sexual abuse was associated with poorer social abilities in a sample of chronic patients suffering from schizophrenia, and subsequent work extended these findings to the vocational and work performance domains (54). Also, participants with histories of maltreatment were significantly more likely to have poorer peer relationships in childhood, and more difficulty in school (77). An increasing number of studies were conducted since, replicating these findings examining mainly the impact of abuse and neglect in small samples of chronic patients with schizophrenia (78), some of them following prospective designs (79, 80). From 2010, larger studies in First-episode of psychosis (FEP) emerged, and interestingly, when the functional level was measured at baseline, in most of them no differences between exposed and non-exposed to abuse were found (23, 81, 82), with still some exceptions (83). Results examining the long-term impact of adversity on functioning are mixed, especially with FEP patients. For instance, Alameda et al. (49) and Alameda et al. (23) showed long lasting detrimental effects on functioning up to 3 years of follow-up as measured with the GAF; while neither Trotta et al. (83) nor Ajnakina et al. (84) did find such differences in GAF at 1 year and 5 years follow-up, respectively. However, the latter showed that living alone was more likely in patients exposed to parental separation (84). Two recent large studies in patients with psychosis (85, 86) confirm the association between exposure to different adversity types and poorer social outcomes after adjusting for a broad range of confounders. These studies highlight other important aspects such as greater effects for non-affective psychoses as compared to affective psychoses (85); the presence of cumulative effects; and a stronger association between emotional trauma and poorer functional outcomes (86).

These disparities between chronic and FEP, and between baseline and follow-up measures can be attributed to different reasons. First, as previously mentioned, the timing of trauma, which has been often underreported (87), can be acting as an important moderator. As far as we know, only two studies have addressed this issue in FEP samples, showing that adversity prior to age 12 is more deleterious and long lasting as compared to when adversity occurs between 12 and 16 (23, 88). Second, observed differences between baseline and follow-up could be due to the progressive development of other mediating or confounding factors during the illness phase and which are not yet present at onset, for example neurocognitive or social cognitive deficits (as aforementioned discussed). Another suggested reason is the potential varying effect of different personality traits (89), and the heterogeneity in outcome measures used across studies, with broad measures such as GAF possibly diluting and masking specific effects between specific subtypes of adversity and functional domains (71).

In summary, heterogeneous evidence suggests a link between exposure to adversity such as abuse, neglect and early parental separation on a range of functioning outcomes, although specific effects need to be better understood. So far, this association seems to be more often present in chronic individuals with psychosis, with the reasons for this yet to be explored; there is some evidence suggesting some cumulative effects (86); and it seems there is a more pervasive effect when exposure occurs earlier (23, 88).

## Is There Evidence of a Mediation Between Adversity and Functional Outcome Through Cognition?

**Figure 1:**
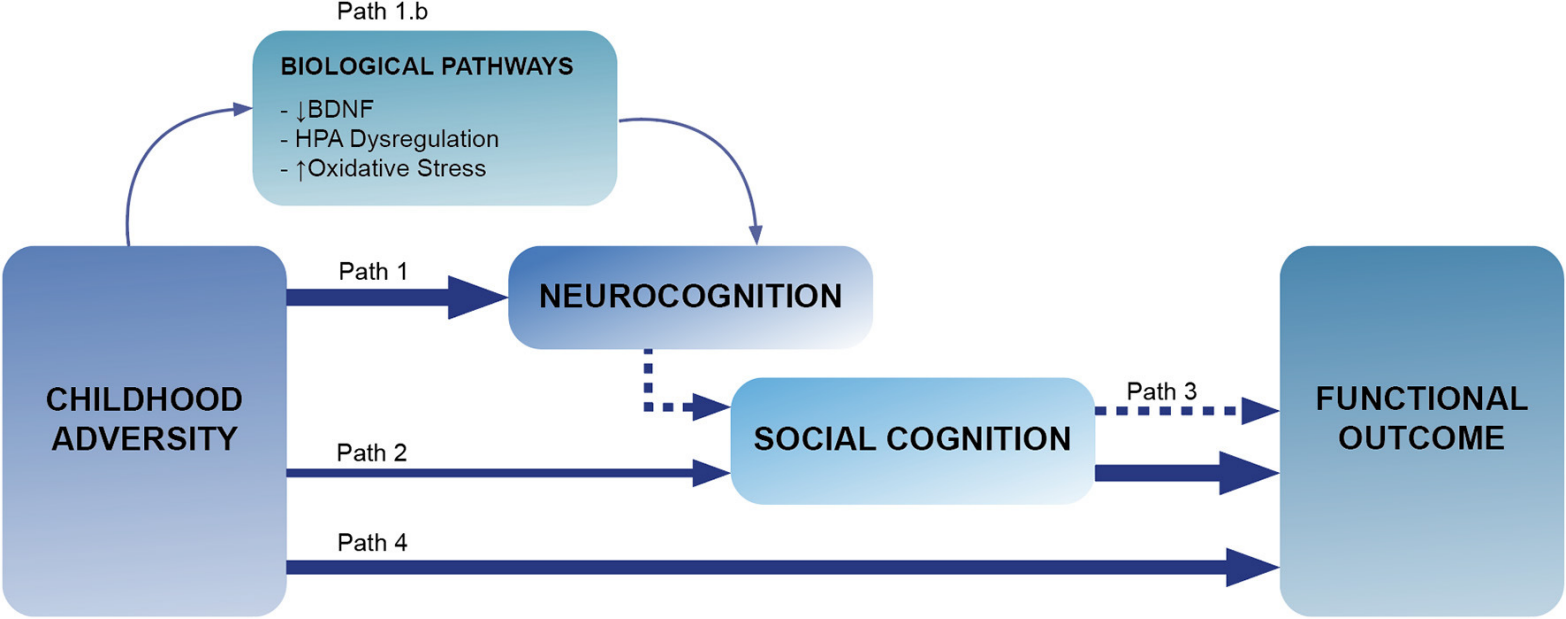
This Figure illustrates the paths explored in this review. Path 1 explores the links between adversity and neurocognition (section Introduction), with path 1.b looking for biological mechanism linking this association (section Adversity and Neurocognition). Path 2 refers to the associations between childhood adversity and social cognition (section Possible Biological Pathways Involved in Cognitive Deficits),with path 3 exploring the links between neurocognition, social cognition and functioning (section Adversity and Social Cognition). Lastly, path 4 corresponds to the links between childhood adversity and functioning (section Interplay Between Neurocognition, Social Cognition, and Functioning in People With Psychosis).

As it has been shown in this review and as it can be illustrated in Figure 1, there is consistent evidence suggesting a link between adversity and functioning; between neurocognition, social cognition and functioning, with suggestions that deficits in neurocognition may precede those in social cognition; and some emerging studies suggesting that adversity also may be associated with social cognitive deficits in patients. Therefore, it seems reasonable to hypothesize that neurocognitive and social cognitive domains may act as mediators between adversity and functioning. However, evidence testing this hypothesis is poor, and the limited evidence available does not support such a hypothesis. Only two studies have tested this in patients with psychosis, both testing social cognition as a mediator (67, 71), and none of them found evidence of mediation. Another study (62) did not test meditation effects, but examined whether deficits in social inference (measured by The Hinting Task) were moderating the effect of adversity on social dysfunctioning measured during 12 months of psychiatric rehabilitation. To the best of our knowledge, no study explored the mediating effect of neurocognition in the adversity-functioning association.

Although a possible mediating role of social cognition between adversity and functioning has been shown in studies conducted in the general population, based on the current evidence, we cannot imply that this is the case in psychosis. However, since only two studies were found testing this hypothesis, we believe that more research is required and that this remains a plausible hypothesis that should be further addressed in future.

## Discussion, Future Directions, and Implications

Most of the research conducted in trying to understand the connections between adversity and psychosis has focused on positive symptoms as the outcome of interest (25). However, as suggested in our review, other domains, also affected by adversity, such as functional decline and cognition, have been notably less well-studied, despite constituting key targets for recovery (7, 90). Considerable effort has been made in trying to understand the determinants of functional outcome considering different aspects of psychopathology and demographic factors (6) or neurocognition and social cognition (7, 14, 74). However, research to date has rarely considered the potential determinant effect of adversity in that equation, and how it can interacts with other important domains, which has been the focus of the current work.

Research presented in this review suggests that when considering the determinants of functional outcome, childhood adversity needs to be considered. As illustrated in Figure 1, our review provides emerging evidence showing a link between adversity and neurocognition (sections Adversity and Neurocognition and Possible Biological Pathways Involved in Cognitive Deficits) and social cognition (section Adversity and Social Cognition) and between childhood adversity and functional impairment (section Adversity and Functional Outcome). Given the links between adversity, neurocognition, social cognition, and functional outcome, there is ground to hypothesize that exposure to adversity may lead to functional impairments in patients through deficits in neurocognition and social cognition, with those in neurocognition preceding the social cognition ones (Figure 1). So far, only two studies have tested the potential mediating effects of social cognition between adversity and functioning (67, 71), where no evidence of such mediation was found. Nevertheless, these pioneer studies were conducted in small samples (141 subjects overall) and require replication. We strongly believe that this is an area that needs to be further explored, and we hypothesize that, despite the so far negative studies, mediation is plausible and should be further investigated.

With regards to biology, addressing studies in the future testing biological mediating mechanisms between adversity and neurocognitive and social cognitive domains will allow exploration of new potential pharmacological targets that could be used as add-on to enhance interventions addressing cognitive deficits (91). In this line, a plausible pathway is related to oxidative stress, which could potentially be corrected with antioxidants, such as N-Acetyl Cysteine (NAC), making it a promising add-on to therapies targeting SC and NC (92). This is of particular interest given evidence showing that traumatized individuals with a better redox status (lower oxidation) showed better cognitive domains as compared with traumatized subjects with higher oxidation and to non-traumatized subjects in terms of cognitive functioning (49). Interestingly, randomized controlled trials in people with psychosis have shown that NAC, a potent antioxidant agent, has shown efficacy in improving cognitive domains in FEP (93) and functioning in chronic patients (94). Supplementing cognitive remediation therapy with antioxidant compounds in people with psychosis with a disrupted redox homeostasis may help to improve their cognition, and subsequently enhance their functional level.

Regarding therapies targeting SC deficits, such as SCIT and SCST, results are still mixed in their potential positive impact on functional outcome from a recent review (7), but the studies included did not take into account the possible moderating effect of exposure to adversity. In light of our findings on the association between adversity and social cognition, we suggest that further studies should test the efficacy of such interventions taking into account exposure to adversity.

## Conclusions

There are solid grounds to suggest that individuals with psychosis and a history of adversity have poorer neurocognitive functions than those without histories of adversity, with also emerging evidence suggesting a link between abuse, neglect, and various social cognitive domains in patients with psychosis. Literature suggests that deficits in neurocognition precedes those in social cognition, and that these domains are particularly deleterious for functioning. To date, no evidence has demonstrated that deficits in cognition may mediate the links between adversity and functioning, but this needs to be further explored as research is still scarce. Different non-competing biological pathways involving the HPA axis, or alterations in the levels of neurotrophic factors and redox dysregulation may be triggered by adversity experiences leading to cognitive alterations in psychoses. These pathways could be differentially expressed across individuals. Selecting patients based on specific biomarker profiles may allow studies to better capture effects between adversity, specific neurocognitive and social cognitive domains, and the ultimate impact on functioning, which can eventually allow specific pharmacological and therapeutic targets to be developed. More research to better understand which subgroups of patients are at greater risk to develop neurocognitive and social cognitive deficits and subsequently poorer functional outcomes is warranted.

## Author Contributions

LA developed the rational and idea of the present manuscript. VR coordinated and aligned the different contributions. All co-authors contributed in parts of the writing and revision of the present review.

## Conflict of Interest

The authors declare that the research was conducted in the absence of any commercial or financial relationships that could be construed as a potential conflict of interest.

## References

[1] Murray CJL Boston MA. The Global Burden of Disease: A Comprehensive Assessment of Mortality and Disability from Diseases, Injuries, and Risk Factors in 1990 and Projected to 2020 Edited by Library of Congress Cataloging-in-Publication (CIP). (1996). Available at: https://apps.who.int/iris/bitstream/handle/10665/41864/0965546608_eng.pdf (accessed August 12, 2020).

[2] Jääskeläinen E Juola P Hirvonen N Mcgrath JJ Saha S Isohanni M. A systematic review and meta-analysis of recovery in schizophrenia. Schizophr Bull. (2013) 39:1296–306. 10.1093/schbul/sbs130 23172003 PMC3796077

[3] Cornblatt BA Carrión RE Addington J Seidman L Walker EF Cannon TD. Risk factors for psychosis: impaired social and role functioning. Schizophr Bull. (2012) 38:1247–57. 10.1093/schbul/sbr136 22080497 PMC3494064

[4] Rössler W Joachim Salize H Van Os J Riecher-Rössler A. Size of burden of schizophrenia and psychotic disorders. Eur Neuropsychopharmacol. (2005) 15:399–409. 10.1016/j.euroneuro.2005.04.009 15925493

[5] Menezes NM Arenovich T Zipursky RB. A systematic review of longitudinal outcome studies of first-episode psychosis. Psychol Med. (2006) 36:1349–62. 10.1017/S0033291706007951 16756689

[6] Galderisi S Rucci P Kirkpatrick B Mucci A Gibertoni D Rocca P. Interplay among psychopathologic variables, personal resources, context-related factors, and real-life functioning in individuals with schizophrenia a network analysis. JAMA Psychiatry. (2018) 75:396–404. 10.1001/jamapsychiatry.2017.4607 29450447 PMC5875306

[7] Halverson TF Orleans-Pobee M Merritt C Sheeran P Fett AK Penn DL. Pathways to functional outcomes in schizophrenia spectrum disorders: meta-analysis of social cognitive and neurocognitive predictors. Neurosci Biobehav Rev. (2019) 105:212–9. 10.1016/j.neubiorev.2019.07.020 31415864

[8] Morin L Franck N. Rehabilitation interventions to promote recovery from schizophrenia: a systematic review. Front Psychiatry. (2017) 8:100. 10.3389/fpsyt.2017.00100 28659832 PMC5467004

[9] Green MF Kern RS Heaton RK. Longitudinal studies of cognition and functional outcome in schizophrenia: implications for MATRICS. Schizophr Res. 72:41–51. 10.1016/j.schres.2004.09.009 15531406

[10] Green MF Penn DL Bentall R Carpenter WT Gaebel W Gur RC. Social cognition in schizophrenia: an NIMH workshop on definitions, assessment, and research opportunities. Schizophr Bull. (2008) 34:1211–20. 10.1093/schbul/sbm145 18184635 PMC2632490

[11] Sheffield JM Karcher NR Barch DM. Cognitive deficits in psychotic disorders: a lifespan perspective. Neuropsychol Rev. (2018) 28:509–33. 10.1007/s11065-018-9388-2 30343458 PMC6475621

[12] Torio I Bagney A Dompablo M Campillo MJ Garcia-Fernandez L Rodriguez-Torresano J. Neurocognition, social cognition and functional outcome in schizophrenia. Eur J Psychiatry. (2014) 28:201–11. 10.4321/S0213-61632014000400001

[13] Green MF. Cognitive impairment and functional outcome in schizophrenia and bipolar disorder. J Clin Psychiatry. (2006) 67(Suppl. 9):3–8; discussion: 36–42. 16965182

[14] Fett A-KJ Viechtbauer W Dominguez M-G Penn DL van Os J Krabbendam L. The relationship between neurocognition and social cognition with functional outcomes in schizophrenia: a meta-analysis. Neurosci Biobehav Rev. (2011) 35:573–88. 10.1016/j.neubiorev.2010.07.001 20620163

[15] Roberts DL Combs DR Willoughby M Mintz J Gibson C Rupp B. A randomized, controlled trial of Social Cognition and Interaction Training (SCIT) for outpatients with schizophrenia spectrum disorders. Br J Clin Psychol. (2014) 53:281–98. 10.1111/bjc.12044 24417608

[16] Horan WP Kern RS Tripp C Hellemann G Wynn JK Bell M. Efficacy and specificity of Social Cognitive Skills Training for outpatients with psychotic disorders. J Psychiatr Res. (2011) 45:1113–22. 10.1016/j.jpsychires.2011.01.015 21377168 PMC4064828

[17] Rocha NBF Queirós C. Metacognitive and social cognition training (MSCT) in schizophrenia: a preliminary efficacy study. Schizophr Res. (2013) 150:64–8. 10.1016/j.schres.2013.07.057 23962827

[18] Horan WP Green MF. Treatment of social cognition in schizophrenia: current status and future directions. Schizophr Res. (2019) 203:3–11. 10.1016/j.schres.2017.07.013 28712968

[19] Dark F Scott JG Baker A Parker S Gordon A Newman E. Randomized controlled trial of social cognition and interaction training compared to befriending group. Br J Clin Psychol. (2020) 59:384–402. 10.1111/bjc.12252 32515058 PMC7496415

[20] Kurtz MM Gagen E Rocha NBF Machado S Penn DL. Comprehensive treatments for social cognitive deficits in schizophrenia: a critical review and effect-size analysis of controlled studies. Clin Psychol Rev. (2015) 43:80–9. 10.1016/j.cpr.2015.09.003 26437567

[21] Van Os J Marsman A Van Dam D Simons CJP GROUP Investigators. Evidence that the impact of childhood trauma on IQ is substantial in controls, moderate in siblings, and absent in patients with psychotic disorder. Schizophr Bull. (2017) 43:316–24. 10.1093/schbul/sbw177 28177077 PMC5605269

[22] Zielinski DS. Child maltreatment and adult socioeconomic well-being. Child Abus Negl. (2009) 33:666–78. 10.1016/j.chiabu.2009.09.001 19811826

[23] Alameda L Ferrari C Baumann PS Gholam-Rezaee M Do KQ Conus P. Childhood sexual and physical abuse: age at exposure modulates impact on functional outcome in early psychosis patients. Psychol Med. (2015) 45:2727–36. 10.1017/S0033291715000690 26350397

[24] Mansueto G Schruers K Cosci F van Os J Alizadeh BZ Bartels-Velthuis AA. Childhood adversities and psychotic symptoms: the potential mediating or moderating role of neurocognition and social cognition. Schizophr Res. (2019) 206:183–93. 10.1016/j.schres.2018.11.028 30527930

[25] Alameda L Rodriguez V Carr E Aas M Trotta G Marino P. A systematic review on mediators between adversity and psychosis: potential targets for treatment. Psychol Med. (2020) 50:1966–76. 10.1017/S0033291720002421 32744193

[26] Howes OD Murray RM. Schizophrenia: an integrated sociodevelopmental-cognitive model. Lancet. (2014) 383:1677–87. 10.1016/S0140-6736(13)62036-X 24315522 PMC4127444

[27] Nijman SA Veling W van der Stouwe ECD Pijnenborg GHM. Social cognition training for people with a psychotic disorder: a network meta-analysis. Schizophr Bull. (2020) 46:1086–103. 10.1093/schbul/sbaa023 32162658 PMC7505203

[28] McGurk SR Twamley EW Sitzer DI McHugo GJ Mueser KT. A meta-analysis of cognitive remediation in schizophrenia. Am J Psychiatry. (2007) 164:1791–802. 10.1176/appi.ajp.2007.07060906 18056233 PMC3634703

[29] Tyborowska A Volman I Niermann HCM Pouwels JL Smeekens S Cillessen AHN. Early-life and pubertal stress differentially modulate grey matter development in human adolescents. Sci Rep. (2018) 8:9201. 10.1038/s41598-018-27439-5 29907813 PMC6003940

[30] Ruby E Rothman K Corcoran C Goetz RR Malaspina D. Influence of early trauma on features of schizophrenia. Early Interv Psychiatry. (2017) 11:322–33. 10.1111/eip.12239 25808607 PMC4580512

[31] Teicher MH Samson JA Anderson CM Ohashi K. The effects of childhood maltreatment on brain structure, function and connectivity. Nat Rev Neurosci. (2016) 17:652–66. 10.1038/nrn.2016.111 27640984

[32] Aas M Pizzagalli DA Fjaera Laskemoen J Reponen EJ Ueland T Melle I. Elevated hair cortisol is associated with childhood maltreatment and cognitive impairment in schizophrenia and in bipolar disorders. Schizophr Res. (2019) 213:65–71. 10.1016/j.schres.2019.01.011 30660575

[33] Liu D Diorio J Tannenbaum B Caldji C Francis D Freedman A. Maternal care, hippocampal glucocorticoid receptors, and hypothalamic- pituitary-adrenal responses to stress. Science. (1997) 277:1659–62. 10.1126/science.277.5332.1659 9287218

[34] Vargas T Lam PH Azis M Juston Osborne K Lieberman A Mittal VA. Childhood trauma and neurocognition in adults with psychotic disorders: a systematic review and meta-analysis. Schizophr Bull. (2019) 45:1195–208. 10.1093/schbul/sby150 30376115 PMC6811825

[35] Aas M Steen NE Agartz I Aminoff SR Lorentzen S Sundet K. Is cognitive impairment following early life stress in severe mental disorders based on specific or general cognitive functioning? Psychiatry Res. (2012) 198:495–500. 10.1016/j.psychres.2011.12.045 22472845

[36] Mørkved N Johnsen E Kroken RA Gjestad R Winje D Thimm J. Does childhood trauma influence cognitive functioning in schizophrenia? The association of childhood trauma and cognition in schizophrenia spectrum disorders. Schizophr Res Cogn. (2020) 21:100179. 10.1016/j.scog.2020.100179 32461919 PMC7240182

[37] McGrath JJ McLaughlin KA Saha S Aguilar-Gaxiola S Al-Hamzawi A Alonso J. The association between childhood adversities and subsequent first onset of psychotic experiences: a cross-national analysis of 23 998 respondents from 17 countries. Psychol Med. (2017) 47:1230–45. 10.1017/S0033291716003263 28065209 PMC5590103

[38] Andersen SL Teicher MH. Stress, sensitive periods and maturational events in adolescent depression. Trends Neurosci. (2008) 31:183–91. 10.1016/j.tins.2008.01.004 18329735

[39] Andersen SL Tomada A Vincow ES Valente E Polcari A Teicher MH. Preliminary evidence for sensitive periods in the effect of childhood sexual abuse on regional brain development. J Neuropsychiatry Clin Neurosci. (2008) 20:292–301. 10.1176/jnp.2008.20.3.292 18806232 PMC4270804

[40] McEwen BS. Sex, stress and the hippocampus: allostasis, allostatic load and the aging process. Neurobiol Aging. (2002) 23:921–39. 10.1016/S0197-4580(02)00027-1 12392796

[41] Ruby E Polito S McMahon K Gorovitz M Corcoran C Malaspina D. Pathways associating childhood trauma to the neurobiology of schizophrenia. Front Psychol Behav Sci. (2014) 3:1–17. 25419548 PMC4236311

[42] Faravelli C Mansueto G Palmieri S Lo Sauro C Rotella F Pietrini F. Childhood adversity, cortisol levels, and psychosis. J Nerv Ment Dis. (2017) 205:574–9. 10.1097/NMD.0000000000000699 28598957

[43] Pruessner M Bechard-Evans L Pira S Joober R Collins DL Pruessner JC. Interplay of hippocampal volume and hypothalamus-pituitary-adrenal axis function as markers of stress vulnerability in men at ultra-high risk for psychosis. Psychol Med. (2017) 47:471–83. 10.1017/S0033291716002658 27774914

[44] Aas M Dieset I Mørch R Steen NE Hope S Reponen EJ. Reduced brain-derived neurotrophic factor is associated with childhood trauma experiences and number of depressive episodes in severe mental disorders. Schizophr Res. (2018) 205:45–50. 10.1016/j.schres.2018.08.007 30126813

[45] Lin CC Huang TL. Brain-derived neurotrophic factor and mental disorders. Biomed J. (2020) 43:134–42. 10.1016/j.bj.2020.01.001 32386841 PMC7283564

[46] Aas M Haukvik UK Djurovic S Tesli M Athanasiu L Bjella T. Interplay between childhood trauma and BDNF val66met variants on blood BDNF mRNA levels and on hippocampus subfields volumes in schizophrenia spectrum and bipolar disorders. J Psychiatr Res. (2014) 59:14–21. 10.1016/j.jpsychires.2014.08.011 25246365

[47] Mondelli V Cattaneo A Murri MB Forti M Di Handley R Hepgul N. Stress and inflammation reduce brain-derived neurotrophic factor expression in first-episode psychosis: a pathway to smaller hippocampal volume. J Clin Psychiatry. (2011) 72:1677–84. 10.4088/JCP.10m06745 21672499 PMC4082665

[48] Aas M Haukvik UK Djurovic S Bergmann Ø Athanasiu L Tesli MS. BDNF val66met modulates the association between childhood trauma, cognitive and brain abnormalities in psychoses. Prog Neuropsychopharmacol Biol Psychiatry. (2013) 46:181–8. 10.1016/j.pnpbp.2013.07.008 23876786

[49] Alameda L Fournier M Khadimallah I Griffa A Cleusix M Jenni R. Redox dysregulation as a link between childhood trauma and psychopathological and neurocognitive profile in patients with early psychosis. Proc Natl Acad Sci USA. (2018) 115:12495–500. 10.1073/pnas.1812821115 30455310 PMC6298080

[50] Green MF Leitman DI. Social cognition in schizophrenia. Schizophr Bull. (2008) 34:670–2. 10.1093/schbul/sbn045 18495642 PMC2632454

[51] Bora E Eryavuz A Kayahan B Sungu G Veznedaroglu B. Social functioning, theory of mind and neurocognition in outpatients with schizophrenia; mental state decoding may be a better predictor of social functioning than mental state reasoning. Psychiatry Res. (2006) 145:95–103. 10.1016/j.psychres.2005.11.003 17074402

[52] Green MF Olivier B Crawley JN Penn DL Silverstein S. Social cognition in schizophrenia: recommendations from the measurement and treatment research to improve cognition in schizophrenia new approaches conference. Schizophr Bull. (2005) 31:882–7. 10.1093/schbul/sbi049 16135561

[53] Toomey R Schuldberg D Corrigan P Green MF. Nonverbal social perception and symptomatology in schizophrenia. Schizophr Res. (2002) 53:5383–91. 10.1016/s0920-9964(01)00177-3 11728841

[54] Lysaker PH Nees MA Lancaster RS Davis LW. Vocational function among persons with schizophrenia with and without history of childhood sexual trauma. J Trauma Stress. (2004) 17:435–8. 10.1023/B:JOTS.0000048957.70768.b9 15633923

[55] Macbeth A Gumley A Schwannauer M Carcione A Fisher R McLeod HJ. Metacognition, symptoms and premorbid functioning in a First Episode Psychosis sample. Compr Psychiatry. (2014) 55:268–73. 10.1016/j.comppsych.2013.08.027 24262130

[56] Fisher HL Schreier A Zammit S Maughan B Munafò MR Lewis G. Pathways between childhood victimization and psychosis-like symptoms in the ALSPAC birth cohort. Schizophr Bull. (2013) 39:1045–55. 10.1093/schbul/sbs088 22941743 PMC3756772

[57] Gibson LE Reeves LE Cooper S Olino TM Ellman LM. Traumatic life event exposure and psychotic-like experiences: a multiple mediation model of cognitive-based mechanisms. Schizophr Res. (2019) 205:15–22. 10.1016/j.schres.2018.02.005 29463456 PMC6098745

[58] Rokita KI Dauvermann MR Donohoe G. Early life experiences and social cognition in major psychiatric disorders: a systematic review. Eur Psychiatry. (2018) 53:123–33. 10.1016/j.eurpsy.2018.06.006 30144982

[59] Aas M Kauppi K Brandt CL Tesli M Kaufmann T Steen NE. Childhood trauma is associated with increased brain responses to emotionally negative as compared with positive faces in patients with psychotic disorders. Psychol Med. (2017) 47:669–79. 10.1017/S0033291716002762 27834153

[60] Aydin O Balikci K Tas C Aydin PU Danaci AE Brüne M. The developmental origins of metacognitive deficits in schizophrenia. Psychiatry Res. (2016) 245:15–21. 10.1016/j.psychres.2016.08.012 27526312

[61] Brañas A Lahera G Barrigón ML Canal-Rivero M Ruiz-Veguilla M. Effects of childhood trauma on facial recognition of fear in psychosis. Rev Psiquiatr Salud Ment. (2019). 10.1016/j.rpsm.2019.01.005. [Epub ahead of print]. 35144915

[62] Choi KH Davidson C Spaulding WD. Social cognition moderates the influence of child physical abuse on inpatient psychiatric rehabilitation. J Nerv Ment Dis. (2011) 199:465–70. 10.1097/NMD.0b013e3182214255 21716060

[63] Barnett D Manly J Cicchetti D. Child Abuse, Child Development, and Social Policy. Norwood, NJ: Ablex Publishing Corporation (1993).

[64] Garcia M Montalvo I Creus M Cabezas Á Solé M Algora MJ. Sex differences in the effect of childhood trauma on the clinical expression of early psychosis. Compr Psychiatry. (2016) 68:86–96. 10.1016/j.comppsych.2016.04.004 27234188

[65] Kilian S Asmal L Chiliza B Olivier M Phahladira L Scheffler F. Childhood adversity and cognitive function in schizophrenia spectrum disorders and healthy controls: evidence for an association between neglect and social cognition. Psychol Med. (2018) 48:2186–93. 10.1017/S0033291717003671 29268811

[66] Lysaker PH Gumley A Brüne M Vanheule S Buck KD Dimaggio G. Deficits in the ability to recognize one's own affects and those of others: Associations with neurocognition, symptoms and sexual trauma among persons with schizophrenia spectrum disorders. Conscious Cogn. (2011) 20:1183–92. 10.1016/j.concog.2010.12.018 21269841

[67] Palmier-Claus J Berry K Darrell-Berry H Emsley R Parker S Drake R. Childhood adversity and social functioning in psychosis: exploring clinical and cognitive mediators. Psychiatry Res. (2016) 238:25–32. 10.1016/j.psychres.2016.02.004 27086207

[68] Rokita KI Dauvermann MR Mothersill D Holleran L Holland J Costello L. Childhood trauma, parental bonding, and social cognition in patients with schizophrenia and healthy adults. J Clin Psychol. (2020) 77:241–53. 10.1002/jclp.23023 32783219

[69] Schalinski I Teicher MH Carolus AM Rockstroh B. Defining the impact of childhood adversities on cognitive deficits in psychosis: an exploratory analysis. Schizophr Res. (2018) 192:351–6. 10.1016/j.schres.2017.05.014 28576548

[70] Trauelsen AM Gumley A Jansen JE Pedersen MB Nielsen HGL Haahr UH. Does childhood trauma predict poorer metacognitive abilities in people with first-episode psychosis? Psychiatry Res. (2019) 273:163–70. 10.1016/j.psychres.2019.01.018 30641347

[71] Weijers J Fonagy P Eurelings-Bontekoe E Termorshuizen F Viechtbauer W Selten JP. Mentalizing impairment as a mediator between reported childhood abuse and outcome in nonaffective psychotic disorder. Psychiatry Res. (2018) 259:463–9. 10.1016/j.psychres.2017.11.010 29145104

[72] Pinkham AE Harvey PD Penn DL. Social cognition psychometric evaluation: results of the final validation study. Schizophr Bull. (2018) 44:737–48. 10.1093/schbul/sbx117 28981848 PMC6007629

[73] Buck BE Pinkham AE Harvey PD Penn DL. Revisiting the validity of measures of social cognitive bias in schizophrenia: additional results from the Social Cognition Psychometric Evaluation (SCOPE) study. Br J Clin Psychol. (2016) 55:441–54. 10.1111/bjc.12113 27168196 PMC5970366

[74] Couture SM Penn DL Roberts DL. The functional significance of social cognition in schizophrenia: a review. Schizophr Bull. (2006) 32:44–63. 10.1093/schbul/sbl029 16916889 PMC2632537

[75] Vaskinn A Sundet K Friis S Simonsen C Birkenaes IB Jónsdóttir H. Emotion perception and learning potential: mediators between neurocognition and social problem-solving in schizophrenia? J Int Neuropsychol Soc. (2008) 14:279–88. 10.1017/S1355617708080314 18282325

[76] Addington J Girard TA Christensen BK Addington D. Social cognition mediates illness-related and cognitive influences on social function in patients with schizophrenia-spectrum disorders. J Psychiatry Neurosci. (2010) 35:49–54. 10.1503/jpn.080039 20040246 PMC2799504

[77] Schenkel LS Spaulding WD Dilillo D Silverstein SM. Histories of childhood maltreatment in schizophrenia: relationships with premorbid functioning, symptomatology, and cognitive deficits. Schizophr Res. (2005) 76:273–86. 10.1016/j.schres.2005.03.003 15949659

[78] Gil A Gama CS de Jesus DR Lobato MI Zimmer M Belmonte-de-Abreu P. The association of child abuse and neglect with adult disability in schizophrenia and the prominent role of physical neglect. Child Abus Negl. (2009) 33:618–24. 10.1016/j.chiabu.2009.02.006 19818499

[79] Davidson G Shannon C Mulholland C Campbell J. A longitudinal study of the effects of childhood trauma on symptoms and functioning of people with severe mental health problems. J Trauma Dissociation. (2009) 10:57–68. 10.1080/15299730802485169 19197712

[80] Lysaker PH Beattie NL Strasburger AM Davis LW. Reported history of child sexual abuse in schizophrenia. J Nerv Ment Dis. (2005) 193:790–5. 10.1097/01.nmd.0000188970.11916.76 16319700

[81] Conus P Cotton S Schimmelmann BG McGorry PD Lambert M. Pretreatment and outcome correlates of sexual and physical trauma in an epidemiological cohort of first-episode psychosis patients. Schizophr Bull. (2010) 36:1105–14. 10.1093/schbul/sbp009 19386579 PMC2963050

[82] Stain HJ Brønnick K Hegelstad WT V Joa I Johannessen JO Langeveld J. Impact of interpersonal trauma on the social functioning of adults with first-episode psychosis. Schizophr Bull. (2014) 40:1491–8. 10.1093/schbul/sbt166 24282322 PMC4193690

[83] Trotta A Murray RM David AS Kolliakou A O'Connor J Di Forti M. Impact of different childhood adversities on 1-year outcomes of psychotic disorder in the genetics and psychosis study. Schizophr Bull. (2016) 42:464–75. 10.1093/schbul/sbv131 26373540 PMC4753600

[84] Ajnakina O Trotta A Forti M Di Stilo SA Kolliakou A Gardner-Sood P. Different types of childhood adversity and 5-year outcomes in a longitudinal cohort of first-episode psychosis patients. Psychiatry Res. (2018) 269:199–206. 10.1016/j.psychres.2018.08.054 30153597

[85] Turner S Harvey C Hayes L Castle D Galletly C Sweeney S. Childhood adversity and clinical and psychosocial outcomes in psychosis. Epidemiol Psychiatr Sci. (2019) 29:e78. 10.1017/S2045796019000684 31839014 PMC8061294

[86] Hjelseng IV Vaskinn A Ueland T Lunding SH Reponen EJ Steen NE. Childhood trauma is associated with poorer social functioning in severe mental disorders both during an active illness phase and in remission. Schizophr Res. (2020) 10.1016/j.schres.2020.03.015. [Epub ahead of print]. 32222348

[87] Cotter J Yung AR. Exploring the impact of adverse childhood experiences on symptomatic and functional outcomes in adulthood: advances, limitations and considerations. Ir J Psychol Med. (2017) 35:5–7. 10.1017/ipm.2017.53 30115206

[88] Alameda L Golay P Baumann PS Progin P Mebdouhi N Elowe J. Mild depressive symptoms mediate the impact of childhood trauma on long-term functional outcome in early psychosis patients. Schizophr Bull. (2017) 43:1027–35. 10.1093/schbul/sbw163 27884931 PMC5581905

[89] Boyette L-L Van Dam D Meijer C Velthorst E Cahn W De Haan L. Personality compensates for impaired quality of life and social functioning in patients with psychotic disorders who experienced traumatic events. Schizophr Bull. (2014) 40:1356–65. 10.1093/schbul/sbu057 24771304 PMC4193722

[90] Penn DL Sanna LJ Roberts DL. Social cognition in schizophrenia: an overview. Schizophr Bull. (2008) 34:408–11. 10.1093/schbul/sbn014 18375928 PMC2632430

[91] Choi KH Til W Kurtz MM. Adjunctive pharmacotherapy for cognitive deficits in schizophrenia: meta-analytical investigation of efficacy. Br J Psychiatry. (2013) 203:172–8. 10.1192/bjp.bp.111.107359 23999481 PMC3759029

[92] Schmidt SJ Hurlemann R Schultz J Wasserthal S Kloss C Maier W. Multimodal prevention of first psychotic episode through N-acetyl-1-cysteine and integrated preventive psychological intervention in individuals clinically at high risk for psychosis: protocol of a randomized, placebo-controlled, parallel-gro. Early Interv Psychiatry. (2019) 13:1404–15. 10.1111/eip.12781 30784233

[93] Conus P Seidman LJ Fournier M Xin L Cleusix M Baumann PS. N-acetylcysteine in a double-blind randomized placebo-controlled trial: toward biomarker-guided treatment in early psychosis. Schizophr Bull. (2018) 44:317–27. 10.1093/schbul/sbx093 29462456 PMC5815074

[94] Berk M Copolov D Dean O Lu K Jeavons S Schapkaitz I. N-acetyl cysteine as a glutathione precursor for schizophrenia-a double-blind, randomized, placebo-controlled trial. Biol Psychiatry. (2008) 64:361–8. 10.1016/j.biopsych.2008.03.004 18436195

